# Abstracts

**DOI:** 10.1155/S1463924683000048

**Published:** 1983


### Abstracts

### Page 9

### Application of a microcomputer system in an infra-red laboratory

M. J. Adams and I. Black

A personal microcomputer can provide inexpensive and powerful computing facilities for data-logging and data-processing in the analytical laboratory. An example is described of the use of an Apple microcomputer in an infra-red laboratory. The computer system is interfaced to dispersive and Fourier spectrometers and extensive data-manipulation software has been produced. The system is compared with instrument manufacturers' dedicated microcomputers.

### Application d'un système à miniordinateur dans un laboratoire pour infrarouge

M. J. Adams and I. Black

Un miniordinateur personnel procure un moyen bon marché et puissant pour l'acquisition des données et leur traitement dans le laboratoire analytique. L'utilisation d'un miniordinateur Apple dans un laboratoire pour infrarouge est décrite. Le système d'ordinateurs est connecté à des spectromètres dispersifs et Fourier et un software puissant pour traitement de données a été produit. Le système est comparé à des miniordinateurs dédiés offerts par les producteurs d'instruments.

### Applikation eines Mikrocomputer-Systems in einem Infrarotlabor

M. J. Adams and I. Black

Ein Mikrocomputer ist eine billige und leistungsfähige Möglichkeit zur Datenerfassung und Datenverarbeitung im analytischen Labor. Ein Beispiel der Verwendung eines Apple Mikrocomputer in einem Infrarotlabor ist näher beschrieben. Das Computersystem ist mit Dispersions- und Fourier-Spektrometern gekoppelt, und es wurde umfassende Datenmanipulationssoftware erstellt. Das System wird mit fest eingesetzten Rechnern von Instrumenten-Herstellern verglichen.

---

### Page 14

### A microcomputer data-acquisition and cumulative reporting system for oestrogen and creatinine continuous-flow analysis

D. Keating *et al.*

Control and data handling of an automatic urinary oestrogen and creatinine analyser has been effected using a microcomputer-based system. The system was designed for use with a two-channel continuous-flow analyser. Two microcomputers are used. The first detects peaks and stores the results. Data is transmitted over a serial link to a second microcomputer which performs the data processing. Cumulative results are printed in tabular and graphical form.

### Acquisition de données par microordinateur et système de compte rendu cumulatif pour le dosage de l'oestrogène et la créatinine

D. Keating *et al.*

Le contrôle et le traitement de données d'un analyseur automatique pour l'oestrogène et la créatinine urinaire a été effectué en utilisant un système basé sur microordinateur. Le système a été conçu pour l'utilisation avec un analyseur a flux continu sur deux canaux. Deux microordinateurs ont été utilisés. Le premier détecte des pics et mémorise les résultats. Les données sont transférées par interface sérielle à un second micro-ordinateur qui, lui, traite les données. Les résultats cumulatifs sont imprimés sous forme graphique et en tableaux.

### Ein Mikrocomputer-Datenerfassungs und kumulatives Protokollier-System für die Analyse von Oestrogen und Kreatinin im kontinuierlichen Durchfluss

D. Keating *et al.*

Mit einem Mikrocomputersystem wurde die Steuerung und Datenerfassung eines automatischen Harnoestrogen- und Kreatinin-Analysators erzielt. Das System war für einen 2-Kanal Durchflussanalysator konzipiert. Zwei Mikrocomputer werden verwendet. Der erste erfasst die Peaks und speichert die Resultate. Die Daten werden über eine serielle Verbindung einem zweiten Mikrocomputer übermittelt, welcher die Datenverarbeitung vollzieht. Kumulative resultate werden in Tabellenform und graphisch ausgedruckt.

---

### Page 18

### The use of a microcomputer system for peak recognition, data processing and representation in continuous-flow analysis

H. Baadenhuijsen and Th. Zelders

The authors describe a stand-alone readout interface for a continuous-flow analyser, based on an Apple II hardware configuration with a program written in BASIC. The system functions in a routine clinical laboratory environment, where high demands are set on the rapid availability of the patient data to be reported. Therefore the system was designed in such a manner that no restrictions are encountered either for the run length or for the exact location of the calibration series within each run. As soon as the peak maximum has reached the colorimeter detector, the result is printed out as a concentration value. Throughout the running time, the system is capable of monitoring possible deviations of the sample and rinse frequency. The results of each run, together with some basic statistics, are recorded on floppy disc in case they are wanted later for retrospective trend-analysis studies. The system shows a great flexibility in that it can be easily reprogrammed for other chemistries by relatively inexperienced laboratory technicians. A complete listing of the programs is available upon request from the first author.

**L'utilisation d'un système à mini-ordinateur pour la reconnaissance de pics, pour le traitement de données et le compte-rendu dans l'analyse à flux continu**

H. Baadenjuijsen and Th. Zelders

Une interface de lecture pour un analyseur à flux continu basé sur un ordinateur Apple II avec un programme écrit en BASIC est décrite. Le système est utilisé dans un laboratoire clinique de routine où les données des malades doivent être rapidement accessibles. C'est pourquoi le système a été conçu de manière que ni la longueur d'une mesure ou la location exacte de la série de calibration n'est restreinte. Dès que le maximum du pic a atteint le détecteur colorimètrique, le résultat est imprimé en unité de concentration. Pendant la mesure, le système est capable de contrôler des déviations possibles de la fréquence de l'échantillon et du rinçage. Les résultats de chaque mesure ainsi que certaines évaluations statistiques sont enregistrés sur floppy disc pour des études rétrospectives ultérieures. Le système montre une grande flexibilité en ce qu'il peut être facilement reprogrammé pour d'autres problèmes chimiques par des techniciens de laboratoire relativement peu qualifiés. Un listing complet des programmes peut être obtenu sur demande du premier auteur.

**Die Verwendung eines Mikrocomputersystems für die Erkennung von Kurvenspitzen, Datenverarbeitung und Datendarstellung in einem Durchflussanalysator**

H. Baadenhuijsen and Th. Zelders

Ein separates Ausleseinterface für einen Durchflussanalysator wird beschrieben, dass auf Apple II Hardware und einem BASIC Programme basiert. Das System arbeitet in einem klinischen Routinelaboratorium, wo hohe Anforderungen an die rasche Verfügbarkeit der Patientendaten gestellt werden. Deshalb wurde das System so ausgelegt, dass keine Einschränkungen vorhanden sind in Bezug auf die Länge der Serie oder die genaue Positionierung der Kalibrationsserien innerhalb einer Serie. Sobald das Kurvenmaximum den Kolorimeterdetektor erreicht hat, wird das Resultat in Konzentrationseinheiten ausgedruckt. Während des ganzen Betriebes ist das System fähig, mögliche Abweichungen der Proben und Waschfrequenz zu überwachen. Die Resultate jeder Serie, zusammen mit einigen statischen Daten, werden auf eine Floppy-Disk ausgegeben für den Fall, dass sie später für retrospektive Trendanalysen benötigt werden. Das System ist sehr flexibel in Bezug auf die leichte Umprogrammierung durch unerfahrenes Laborpersonal wegen anderer Chemie. Ein vollständiger Ausdruck der Programme ist auf Verlangen vom ersten Autor erhältlich.

---

**Page 22**

**An evaluation of the Ames Seralyzer**

P. M. S. Clark and P. M. G. Broughton

An evaluation of the Ames Seralyzer for the measurement of serum glucose, urea and urate has shown that results agreed well with those obtained with the SMA 12/60 analyser, although precision was not so good. Results were less satisfactory for high values, which require additional dilution of the sample, and this appears to introduce some non-linearity. Results on quality-control sera for all three analytes were on average about 8% different from those found by comparable manual methods. The analytical range was limited, particularly for glucose: values in the hypoglycaemic range could not be measured. The instrument is restricted to serum, and fluoride cannot be used as a preservative. Although simple to use, some skill was required in applying the sample to the reagent strip and the instrument was labour-intensive. The cost per test is relatively high with small work-loads.

The instrument may be useful in situations where few samples need to be analysed quickly by a semi-skilled operator. However, for glucose measurements, cheaper alternatives are available.

**Une évaluation du Seralyzer Ames**

P. M. S. Clark and P. M. G. Broughton

Une évaluation du Seralyzer Ames pour l'analyse de la glucose, l'urée et l'urate a montré que les réultats correspondaient bien avec ceux obtenus par l'analyseur SMA 12/60, mais que la précision était moins bonne. Les résultats étaient moins bons pour les valeurs élevées qui demandent une dilution additionnelle de l'échantillon ce qui semble introduire une certaine non-linéarité. Les résultats sur des sérums de contrôle de qualité pour les 3 substances variaient en moyenne de 8% de ceux trouvés par des méthodes manuelles comparables. Le domaine analytique était limité en particulier pour la glucose: les valeurs dans la plage hypoglycémique n'ont pas pu être mesurées. L'instrument est limité au sérum et le fluorure ne peut pas être utilisé comme préservatif. Bien que simple à utiliser, une certaine habilité était demandée pour appliquer l'échantillon et l'instrument demande beaucoup de travail. Le coût par test est relativement élevé pur des petites séries. L'instrument peut être pratique dans des situations où peu d'échantillons doivent être analysés rapidement par une personne semi-qualifiée. Par contre, pour des mesures de glucose des alternatives meilleur marché existent.

**Eine Beurteilung des Ames Seralyzer**

P. M. S. Clark and P. M. G. Broughton

Die Evaluation des Ames Seralyzer für die Bestimmung von Serumglukose, Harnstoff und Urat ergab eine gute Uebereinstimmung der Resultate mit denjenigen, die mit dem SMA 12/60 Analysator erhalten wurden. Allerdings war die Präzision nicht so gut. Die Resultate waren für hohe Werte, welche eine zusätzliche Verdünnung der Probe verlangen weniger zufriedenstellend. Diese scheint eine leichte Nichtlinearität zu verursachen. Die Resultate für Qualitätskontrollseren aller drei Analyten wichen im Durchschnitt ungefähr 8% von den Werten ab, die mit vergleichbaren manuellen Methoden gefunden werden. Der analytische Bereich speziell für Glukose, war eingeschränkt: Werte im hypoglykämischen Bereich konnten nicht gemessen werden. Das Gerät ist beschränkt auf Serum, und Fluorid kann nicht als räservativ eingesetzt werden. Obwohl einfach zu gebrauchen, benötigt das Gerät einige Geschicklichkeit, um die Probe auf den Reagenzstreifen aufzubringen. Das Instrument ist arbeitsintensiv. Bei geringem Durchsatz sind die Kosten pro Analyse relativ hoch.

Das Instrumet mag nützlich sein in Situationen, wo wenige Proben von einem halbausgebildeten Benützer rasch analysiert werden sollen. Jedoch gibt es für Glukosebestimmungen preisgünstigere Alternativen.

### Page 27

### A dedicated automatic dilutor for the automation of $\alpha_2$ macroglobulin kinetic studies

C. Riley *et al.*

A new method of classifying $\alpha_2$ macroglobulin into one of seven categories has been devised. The manual technique is tedious involving preparation of 25 dilutions of serum and making 25 kinetic measurements. As a first step to automation of this method an automatic diluter is proposed. The paper gives constructional details of the dilutor. This includes two specially constructed narrow-bore syringes 30 cm in length, the pistons of which are driven by screw threads operated by stepping motors. The syringe mechanism and the drive to the container carriage are under microcomputer control. The dilutor prepares the 25 dilutions quickly and accurately. Details are given of the evaluation of the machine.

### Un diluteur automatique pour l'automation des études cinétiques de l'$\alpha_2$ macroglobuline

C. Riley *et al.*

Une nouvelle méthode pour classifier les $\alpha_2$ macroglobulines en une de sept catégories a été développée. La technique manuelle est pénible et demande la préparation de 25 dilutions de sérum et de 25 mesures cinétiques. Comme premier pas vers l'automation de cette méthode un diluteur automatique est proposé. Des détails de construction du diluteur sont décrits. Deux seringues minces spécialement construites de 30 cm de longeur dont les pistons sont activés par un arbre fileté et un moteur pas à pas sont utilisées. Le méchanisme de la seringue et l'activation du récipient sont sous contrôle d'un miniordinateur. Le diluteur prépare les 25 dilutions rapidement et précisément. Des détails sur l'évaluation de cette machine sont donnés.

### Ein speziell automatischer Verdünner für die Automatisierung von $\alpha_2$ Makroglobulin Kinetik-Studien

C. Riley *et al.*

Eine neue Methode zur Klassifizierung von $\alpha_2$ Makroglobulin in eine von sieben Kategorien wurde erstellt. Die manuelle Technik ist langwierig und benötigt zudem eine Vorbereitung von 25 Serumverdünnungen sowie 25 kinetische Messungen. Als erster Schritt, diese Methode zu automatisieren, wird ein automatischer Verdünner vorgeschlagen.

Es werden konstruktive Einzelheiten zum Verdünner beschrieben. Diese beinhalten zwei speziell konstruierte Motorkolbenspritzen von 30 cm Länge, welche von Schrittmotoren über ein Schraubengewinde angetrieben werden. Der Spritzenmechanismus wird durch einen Mikrocomputer gesteuert. Die 25 Verdünnungen werden vom Verdünner schnell und genau vorbereitet. Es werden nähere Angaben zur Beurteilung dieses Gerätes gemacht.

### Page 32

### A stopped-flow/flow-injection system for automation of $\alpha_2$ macroglobulin kinetic studies

C. Riley *et al.*

Kinetic studies on $\alpha_2$ macroglobulin developed by workers at the University of Sussex promise to be of considerable clinical significance. However, the manual technique used so far is tedious and time-consuming—requiring 25 separate kinetic assays. As part of a continuing project to mechanize the technique, a dedicated automatic dilutor has been constructed. The present paper describes the next phase of the development, namely the construction of a stopped-flow, flow-injection kinetic machine. This was assembled largely from readily available components; the one component which had to be constructed—the sampling valve—is described in some detail. The automatic system almost halves the time required for a full assay and correlates well with the manual technique.

### Un système stopped-flow/flow-injection pour l'automation de l'étude des $\alpha_2$-macroglobulines

C. Riley *et al.*

Des études cinétiques développées pour l'$\alpha_2$-macroglobuline à l'Université de Sussex promettent d'être de signification clinique considérable. La technique manuelle utilisée aujourd'hui est ennuyeuse et demande 25 analyses cinétiques différentes et beaucoup de temps. Dans le cadre d'un projet continu de mécanisation un diluteur automatique dédié a déjà été construit. Cette publication présente la prochaine phase du développement c.-à-d. la construction d'une machine pour mesures cinétiques par stopped-flow/flow injection. Celle-ci a été réalisée en utilisant des composantes commerciales; la valve pour échantillonnage qui a dû être construite est décrite en plus de détails. Le système automatique permet de diminuer le temps de moitié et donne une bonne corrélation avec la technique manuelle.

### Ein 'stopped-flow/flow-injection' System für die Automatisierung von $\alpha_2$-Makroglobulinuntersuchungen

C. Riley *et al.*

Die von den Mitarbeitern der Universität Sussex durchgeführten kinetischen Studien an $\alpha_2$-Makroglobulin versprechen von beträchtlicher klinischer Bedeutung zu sein. Die bisher verwendete manuelle Technik ist jedoch mühsam und zeitraubend, bedingt sie doch 25 verschiedene kinetische Analysen. Als Teil eines laufenden Projektes zur Mechanisierung der Technik wurde ein zweckgebundener

Verdünnungsautomat konstruiert. Die vorliegende Arbeit beschreibt die nächste Phase der Entwicklung, nämlich der Konstruktion eines 'stopped-flow, flow-injection kinetic' Gerätes. Dieses setzt sich weitgehend aus leicht erhältlichen Komponenten zusammen; die eine Komponente, welche konstruiert werden musste, nämlich das Probenziehventil, wird im Detail beschrieben. Das automatisierte System vermag die erforderliche Zeit für eine volle Analyse fast zu halbieren und korreliert sehr gut mit der manuellen Technik.

---

### Page 36

### Automated pre-column derivatization and its application to amino-acid analysis using high-performance liquid chromatography

D. C. Turnell and J. D. H. Cooper

An automated pre-column derivatization system for sample preparation and injection onto a high-performance liquid chromatograph (HPLC) is described. The o-phthalaldehyde/2-mercaptoethanol derivatization of amino-acids is used to illustrate this application. The system uses minimal sample volumes and the overall coefficients of variation for the technique were found to range from 0·9 to 2·2%.

### Dérivatisation avant la colonne automatisée et son application à l'analyse des acides aminés utilisant la chromatographie liquide à haute performance

D. C. Turnell and J. D. H. Cooper

Un système automatique pour dérivatisation avant colonne pour la préparation d'échantillons et l'injection dans un chromatographe liquide à haute performance (HPLC) est décrit. La dérivatisation des acides aminés avec la o-phthalaldehyde/2-mercaptoéthanole est utilisée pour illustrer cette application. Ce système utilise un volume d'échantillon minimal et les coéfficients de variation pour cette technique se trouvent dans la plage de 0,9 à 2,2%.

### Automatisierte Derivatisierung vor der Kolonne und ihre Anwendung auf die Aminosäuren-Analyse mit der Hochleistungs-Flüssigchromatographie

D. C. Turnell and J. D. H. Cooper

Ein automatisiertes Derivatisierungssystem für die Probenvorbereitung vor der Kolonne und Einspritzung in einen Hochleistungs-Flüssigchromatographen (HPLC) wird beschrieben. Mittels der o-Phthalaldehyd/2-Merkaptoäthanol-Derivatisierung wird diese Anwendung dargestellt. Das System benützt minimale Probenvolumina, und der Variationskoeffizient über alles dieser Technik bewegt sich im Bereich 0,9–2,2%.

---

### Page 40

### Measurement of ionized calcium using the Nova 2 analyser

P. Ferreira and B. E. Jacobson

The performance characteristics of the Nova 2 ionized calcium analyser were evaluated with respect to its practical use in the routine clinical laboratory. The optimum precision was achieved by direct serum sampling from full vacutainers and when each serum analysis was preceded by an aqueous standard. The instrument was generally reliable, but the major drawback was that results obtained with different electrodes were variable. Ionic interferences appeared to be minimal, except for a marked negative interference by magnesium. A negative bias was observed due to the use of carbon dioxide permeable silicone rubber tubing. Serum protein appeared to cause a positive interference in ionized calcium measurements and a significant correlation was observed between ionized calcium and total protein. Ionized calcium correlated well with total calcium but the correlation coefficient was lowered by attempting to correct the total calcium for total protein or albumin levels. Ionized calcium in post-plasmapheresis patient sera fell proportionally more than total calcium, explained mainly by the complexing of calcium ions by citrate anticoagulant. Patients on total parenteral nutrition had normal or near normal ionized calcium levels.

### Mesure du calcium ionisé utilisant l'analysateur Nova 2

P. Ferreira and B. E. Jacobson

La performance de l'analisateur Nova 2 pour calcium ionisé a été évaluée en vue de l'utilisation pratique dans le laboratoire clinique. La meilleure précision résulte par échantillonnage direct d'un récipient de sérum sous vide et quand chaque analyse de sérum est précédée d'un standard aqueux. L'instrument donne en général des résultats sûrs, mais le principal problème sont les résultats variables quand on utilise différentes électrodes. Les interférences par d'autres ions étaient minimales à l'exception d'une interférence négative due au magnésium. Une déviation négative a été observée à cause de l'utilisation de tubes en silicone perméables pour le $CO_2$. Les protéines de sérum semblent causer une interférence positive dans les mesures du calcium ionisé et une corrélation significative a été observée entre le calcium ionisé et les protéines. Le calcium ionisé donne une bonne corrélation avec le calcium total, mais le coéfficient de corrélation a été abaissé par l'essai de corriger le calcium total pour les niveaux d'albumines ou de protéines totales. Le calcium ionisé pour des malades post-plasma-phérétiques s'abaissa plus que le calcium total ce qui s'explique surtout par la complexation des ions de calcium par l'anticoagulant de citrate. Les malades avec nutrition parentérale complète avaient des niveaux de calcium normaux ou presque normaux.

### Bestimmung von ionisiertem Kalzium mit dem Nova 2 Analysator

P. Ferreira and B. E. Jacobson

Die Leistungsdaten des Nova 2 Analysators für ionisiertes Kalzium wurden in Bezug auf den praktischen

Einsatz in einem klinischen Routinelabor evaluiert. Die optimale Präzision wurde durch direktes Ziehen der Serumprobe aus vollen 'vacutainers', und wenn jeder Serumanalyse ein wässriger Standard voranging, erreicht. Das Instrument wurde im allgemeinen als zuverlässig befunden. Ein Hauptnachteil hingegen war die Tatsache, dass mit verschiedenen Elektroden verschiedene Resultate erhalten wurden. Ausser einer markanten negativen Interferenz durch Magnesium schienen die Ionen-Interferenzen minimal zu sein. Wegen des Gebrauchs von $CO_2$-durchlässigen Silikongummischläuchen wurde eine negative Verschiebung beobachtet. Serumprotein scheint bei Bestimmungen von ionisiertem Kalzium eine positive Interferenz zu bewirken, und es wurde eine signifikante Korrelation zwischen ionisiertem Kalzium und Gesamtprotein beobachtet. Ionisiertes Kalzium korrelierte gut mit Gesamtkalzium, aber der Korrelationskoeffizient wurde erniedrigt, wenn versucht wurde, das Gesamtkalzium wegen des Gesamtproteins oder des Albumins zu korrigieren. Ionisiertes Kalzium in Post-Plasmapheresis-Patientenseren fiel proportional stärker als Gesamtkalzium. Dies wird hauptsächlich durch die Komplexierung der Kalziumionen durch das Zitrat-Antiokoagulant erklärt. Patienten mit vollständig parenteraler Ernährung wiesen einen normalen oder fast normalen Spiegel an ionisiertem Kalzium auf.

### Page 43

### Evaluation of assays for thyroxine, triodotyronine and thyroid-stimulating hormone on the ARIA II automated radioimmunoassay instrument

M. J. Duffy *et al.*

The Aria II automated radioimmunoassay system was used to assay thyroxine, triodotyronine and thyroid-stimulating hormone. Correlation coefficients with established manual methods were: thyroxine, $r=0{\cdot}91$, triodotyronine, $r=0{\cdot}95$ and for thyroid-stimulating hormone, $r=0{\cdot}93$. Within-assay variation using commercial control serum ranged from 2·58 to 6·44% for thyroxine, from 3·27 to 10·7% for triodotyronine and from 8·0 to 15·3% for thyroid-stimulating hormone. Between-assay variation ranged from 4·60 to 7·57% for thyroxine, from 7·52 to 11·7% for triodotyronine and from 8·64 to 14·7% for thyroid-stimulating hormone. Carry-over was not significant for any of the assays. The ARIA II system gives good precision for routine thyroid function tests and at the same time saves a considerable amount of labour.

### Evaluation de l'analyse de la thyroxine, la triodotyronine et de l'hormone stimulant la thyroïde sur l'instrument automatique pour radioimmunoassay ARIA II

M. J. Duffy *et al.*

Le système automatique pour radioimmunoassay ARIA II a été utilisé pour analyser la thyroxine, la triodotyronine et l'hormone stimulant la thyroïde. Les coéfficients de corrélation avec les méthodes manuelles établies sont: thyroxine, $r=0{,}91$, triodotyronine, $r=0{,}95$ et pour l'hormone stimulant la thyroïde $r=0{,}93$. Les mesures de variation en utilisant des sérums de contrôle commerciaux ont donné de 2,58 à 6,44% pour la thyroxine, de 3,27 à 10,7% pour la triodotyronine et de 8,0 à 15,3% pour l'hormone stimulant la thyroïde. Les variations entre différents essais variaient de 4,60 à 7,57% pour la thyroxine, de 7,52 à 11,7% pour la triodotyronine et de 8,54 à 14,7% pour l'hormone stimulant la thyroïde. L'entraînement n'était pas significatif pour aucune des analyses. Nous concluons que le système ARIA II donne une bonne précision pour les contrôles de routine de la fonction de la thyroïde et en même temps permet de réduire le travail.

### Beurteilung von Analysen für die bestimmung von Thyroxin, Triodtyronin und Thyroid-stimulieremden Hormon auf dem ARIA II Gerät für automatisierte Radioimmunoassay RIA

M. J. Duffy *et al.*

Das ARIA II System für automatisierte RIA wurde für die Analyse von Thyroxin, Triodtyronin und Thyroid-stimulierendem Hormon benützt. Die Korrelationscoeffizienten mit etablierten manuellen methoden waren: Thyroxin, $r=0{,}91$, Triodtyronin, $r=0{,}95$ und für Thyroid-stimulierendes Hormon, $r=0{,}93$. Innerhalb einer Analysenserie betrug die Variation bei Benützung eines kommerziellen Kontrollserums 2,58 bis 6,44% für Thyroxin, 3,27 bis 10,7% für Triodtyronin und 8,0 bis 15,3% für Thyroid-stimulierendes Hormon. Zwischen Analysenserien betrug die Variation 4,6 bis 7,57% für Thyroxin, 7,52 bis 11,7% für Triodtyronin und 8,64 bis 14,7% für Thyroid-stimulierendes Hormon. Für keine dieser Analysen war die Verschleppung signifikant. Wir schliessen daraus, das dass ARIA II System gute Präzision für routinemässige Schilddrüsen Funktionsprüfungen ergibt und dabei gleichzeitig in erheblichem Ausmass Arbeit einspart.

